# Advances in Sensor Technologies in the Era of Smart Factory and Industry 4.0 †

**DOI:** 10.3390/s20236783

**Published:** 2020-11-27

**Authors:** Tahera Kalsoom, Naeem Ramzan, Shehzad Ahmed, Masood Ur-Rehman

**Affiliations:** 1 School of Computing, Engineering and Physical Sciences, University of West of Scotland, Paisley PA1 2BE, UK; naeem.ramzan@uws.ac.uk; 2 School of Business and Creative Industries, University of West of Scotland, Paisley PA1 2BE, UK; shehzad.ahmed@uws.ac.uk; 3 James Watt School of Engineering, University of Glasgow, Glasgow G12 8QQ, UK; masood.urrehman@glasgow.ac.uk

**Keywords:** smart factory, internet-of-things, sensors, manufacturing, Industry 4.0

## Abstract

The evolution of intelligent manufacturing has had a profound and lasting effect on the future of global manufacturing. Industry 4.0 based smart factories merge physical and cyber technologies, making the involved technologies more intricate and accurate; improving the performance, quality, controllability, management, and transparency of manufacturing processes in the era of the internet-of-things (IoT). Advanced low-cost sensor technologies are essential for gathering data and utilizing it for effective performance by manufacturing companies and supply chains. Different types of low power/low cost sensors allow for greatly expanded data collection on different devices across the manufacturing processes. While a lot of research has been carried out with a focus on analyzing the performance, processes, and implementation of smart factories, most firms still lack in-depth insight into the difference between traditional and smart factory systems, as well as the wide set of different sensor technologies associated with Industry 4.0. This paper identifies the different available sensor technologies of Industry 4.0, and identifies the differences between traditional and smart factories. In addition, this paper reviews existing research that has been done on the smart factory; and therefore provides a broad overview of the extant literature on smart factories, summarizes the variations between traditional and smart factories, outlines different types of sensors used in a smart factory, and creates an agenda for future research that encompasses the vigorous evolution of Industry 4.0 based smart factories.

## 1. Introduction

With its introduction in Germany in 2011, Industry 4.0 instantly became the focus of a global world that promoted the computerization of manufacturing [1]. Industry 4.0 has revolutionized the manufacturing process, leading to intelligent manufacturing promising self-sufficient manufacturing processes by using machines and devices that communicate with each other through digital connectivity [2,3]. Although most of the design philosophies and technologies of Industry 4.0, such as the internet of things (IoT), cyber-physical systems (CPSs), and artificial intelligence are already in use, most firms lack insight into the wide set of technologies offered by Industry 4.0 that provide devices with seamless connectivity, interoperability, visibility, and intelligence capabilities.

Applications in traditional manufacturing are stand-alone and segregated [4], and lack automated monitoring and control capabilities [5]. There are a series of distinct and independent steps, including marketing, product development, manufacturing, and distribution to customers [1]. As a result, the reuse of systems, and the integration of physical and digital systems, in traditional manufacturing is poor [6]. Some advanced manufacturing strategies, such as intelligent manufacturing, flexible manufacturing, and agile manufacturing have the potential to overcome the drawbacks of traditional manufacturing [7,8,9]. These manufacturing schemes are the pioneers of Industry 4.0 smart manufacturing, where machines and products interact with each other without, or with minimal, human control [4,6].

Manufacturing industry plays a crucial role in the evolution of modern society. Industry 4.0, which is the pioneer of smart factories, has access to various advanced technologies such as big data analytics, artificial intelligence, advanced robotics, 3D printing, and cloud computing [1,3]. The vast implementation of computer numerical control (CNC) and industrial robots has enabled a flexibility in manufacturing systems [10,11,12]; whereas computer-aided design (CAD) and computer-aided processing planning (CAPP) have made computer integrated manufacturing practical [13,14]. The actual uses of the IoT have enabled manufacturers to adopt digital transformations from different prospects, such as efficient productivity, automation, customer focus, competitive advantages, and enhancing the value chain and rapid returns [15,16].

In an attempt to understand the efficient use of the IoT in the manufacturing industry, it is imperative to recognize the different technologies, especially sensors that make the performance of manufacturing firms efficient in using Industry 4.0. By combining everyday objects with connected devices through IoT, it is possible to gather information, analyze it, and create an action that learns from processes. The focal objective of the concept of Industry 4.0 is to characterize highly digitized manufacturing processes, where flow of information amid different devices is controlled in an environment with very limited human intervention [3,5,17]. Cloud-based IoT platforms have the ability to connect the real world with the virtual world, enabling companies to manage IoT device connectivity and flexibility [18]. In addition, the IoT architecture must be flexible enough to operate different wireless protocols and accommodate additions of new sensor inputs (e.g., USB) [19]. This can also be acknowledged in terms of physical flexibility, which can include wearable devices, carry devices, battery usage, etc. [7]. The use of sensors makes this achievable.

Recently, Industry 4.0 based manufacturing processes have attracted a lot of attention from academia [5,6,7,20], with the main focus on different areas such as sustainability, organizational structure, lean manufacturing, product development, and strategic management within the manufacturing industry. Researchers have investigated the relationship between different optimal control models and an Industry 4.0 based smart factory system [9]. Comprehensive work has been done in analyzing the benefits, challenges, and risks involved with implementing smart factories. The majority of the extant literature focuses on the contributions and threats of IoT related to flexibility, transparency, information sharing, connectivity, traceability, and tracking within Industry 4.0. While a lot of research has been carried out with a focus on analyzing the performance, processes, and implementation of Industry 4.0 based smart factories, it has been found that most firms still lack in-depth insight into the difference between traditional and smart factory systems, as well as the wide set of different sensor technologies associated with Industry 4.0. This paper tries to fill this gap by identifying the different sensor technologies of Industry 4.0 available, and identifying the differences between traditional and smart factories. In addition, this paper reviews existing research that has been done on Industry 4.0; therefore, provides a broad overview of the extant literature on Industry 4.0, summarizes the variations between traditional and smart factories, and creates an agenda for future research that encompasses the vigorous evolution of Industry 4.0.

## 2. Smart Factory

Although automation has become a crucial part of the factory, innovative manufacturers have taken the opportunity to take it to a whole new level through the application of IoT and artificial intelligence in production processes. With enhancing the complexity of cyber physical systems, the physical machines and business processes have combined with automation to give rise to complex optimization decisions that were made traditionally by humans [5,21,22]. This has enabled manufacturers to integrate the floor decisions and perceptions with the supply chain, giving birth to what we now call a smart factory. The introduction of mechanical manufacturing equipment marked the first industrial revolution, followed by the development of the mass production of goods [13]. The digital revolution was considered to be the adoption of increased automation and control in manufacturing processes by using electronics and IT [12]. The adoption of IoT in these processes has given rise to the deviation of the centralized factory system to a decentralized system [12,13,14]. This technology has enabled machines and industries to go through a self-optimization and reconfiguration, to adapt their behavior to changes in orders and operating conditions [23,24]. The core of smart factories is the technology that makes data collection possible. This technology includes intelligent sensors, motors, and robotics which are employed on the production and assembly lines of the manufacturing industry [17].

### Smart Factory vs. Traditional Factory

**Figure 1:**
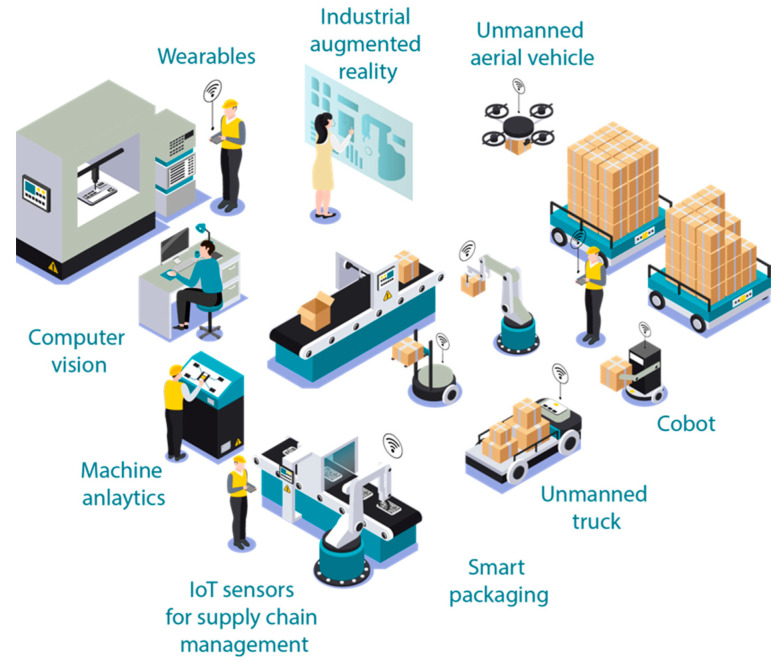
Concept of a smart factory.

In order to meet drastic changes in customer demands, the manufacturing process requires abilities that help in adjusting product type and production capacity, to enable the handling of multiple product varieties [25]. Manufacturing should have adequate functionality, scalability, and connectivity with customers and suppliers to meet such challenges. Traditional factories lack capabilities that allow them to monitor and control automated and complex manufacturing to enable efficient production of customized products [26]. Traditional factories have stand-alone and segregated applications with less integration of the production system, product life cycle, and value chain. Consequently, there is poor reuse of systems and integration between real and virtual systems in a traditional set-up. A general concept of a smart factory can be seen in Figure 1.

The smart factory is an upgrade from old-fashioned automation to a linked and flexible system, and which constitutes a continual data stream through highly connected operations and production systems which can learn and adapt to changing demands [26,27,28]. These factories can assimilate data from physical, operational, and human assets to drive manufacturing, maintenance, inventory tracking, digitization of operations, and other activities in manufacturing systems [29]. The main aim of smart factories is to use intelligent production systems and suitable engineering methods for the successful and interconnected implementation of production facilities [25,30]. It is an engineering system that operates on interconnection, collaboration, and execution. Interconnecting devices in smart factories allow the exchange of information, recognize and assess situations, and integrate the physical world with the digital world, making smart factories adaptive in nature [23,31]. In other words, the smart factory integrates physical and cyber technologies and makes the involved technologies more accurate, enhancing the performance, quality, controllability, management, and transparency of the manufacturing processes. In such a smart factory environment, the manufacturer has the ability to meet customer requirements by changing the production specifications and other settings of the machines at the last minute. This ability is not present in traditional factories [32] (Table 1). The true feature of a smart factory lies in its capability to readjust and evolve along with the growing needs of the organization [32,33]. These needs can be categorized into: changing customer demands, emergence of new markets, development of new products and services, enhanced productive approaches to operations, and use of advanced technologies in maintenance processes [13,34,35]. The ability to tailor and learn from real-time data makes smart factories more receptive and predictive, to avoid operational downtime and other possible failures in processes [12,29].

A smart factory is characterized by four intelligent features:

- Sensors: these are devices that have the ability to self-organize, learn, and maintain environmental information to analyze behaviors and abilities. Therefore, sensors can make decisions that enable them to adjust to changes in the environment.
- Interoperability: through interconnection between different devices, coordination between them can be enhanced, allowing flexibility in configuration protocols of the production system.
- Integration: robots and artificial intelligence (AI) allow smart factories to have a high level of integration among processes. AI, along with the integration of human intellectual capabilities, enables factories to perform analysis and decision making.
- Virtual reality (VR) techniques: as one of the high-level components of smart factories, VR facilitates human–machine integration by virtualizing manufacturing processes using computers, signal processing, animation technology, intelligent reasoning, prediction, and simulation and multimedia technologies.

## 3. Key Sensing Technologies in a Smart Factory

Smart factories are comprised of intelligent machines, devices, and control equipment that monitor vital parameters of the manufacturing processes [5]. These improvements have not only altered the factory floor infrastructure, promoting steady and precise collaboration between machines, but have also altered machinery requirements, increasing demand for reliable sensors [10]. This section gives brief information about the key sensors used in a smart factory.

### 3.1. Passive Sensors

The current manufacturing system is defined by different technologies, however the main technologies used are sensors, actuators, effectors, controllers, and control loops [36]. A vital role is played by sensors in a smart factory, as they collect and implement accurate data into the manufacturing processes to enhance product quality. Sensors are electrical, opto-electrical, or electronic devices consisting of sensitive materials that help to determine the presence of a particular entity or function [37]. In many cases, a physical stimulus is transformed into an electrical signal using sensors, which then can be evaluated and analyzed for making decisions about the operations being carried out [5,36]. Recent developments in sensor technology have enabled manufacturers to control and acquire data like never before.

Sensors can operate either actively or passively [36,38]. When operating actively, a particular physical stimulus is required for the sensor to work. For example, color identification sensors are active as they need visible light to illuminate the object so that the sensor can receive a physical stimulus. In the passive instance, the physical stimulus is already present and does not have to be provided [38]. For example, infrared devices are passive as the stimulus is already being generated from infrared radiation that is linked with the temperature of a body.

Several types of sensors have been developed and used successfully in industrial process control. Smart factories use a variety of sensor types, from basic temperature to humidity monitoring to sophisticated position and product sensing [39,40]. These sensors make manufacturing efficient by helping in advance factory operations, such as moving products, controlling robotic and milling processes, and sensing environmental factors. The main measurement and control parameters in a factory environment are temperature, position, force, pressure, and flow [40].

#### 3.1.1. Temperature Sensors

As temperature directly affects material properties and product quality, it is one of the crucial parameters to be measured and controlled in industrial plants. A temperature sensor is a device that has the ability to collect temperature concerned information from a resource, and then changes it into information that can be understood by another device [41]. These sensors have the ability to measure the thermal characteristics of gases, liquids, and solids. Several temperature sensors have been developed in recent years which can be used in electrically and chemically hostile environments. These sensors can be divided into two groups: (1) low-temperature sensors, with a range of −100 to +400 °C, using sensing materials such as phosphors, semi-conductors, and liquid crystals; and (2) high-temperature sensors with a range of 500 to 2000 °C, based on blackbody radiations [25,40,42]. Table 2 shows the different sub-types of temperature sensor, along with their key features.

#### 3.1.2. Pressure Sensors

Pressure sensors have the ability to capture pressure changes and transform them into an electrical signal, where the applied pressure defines its quantity. These are electro-mechanical devices that identify force in gases or liquids and provide control signals to display devices [25,37,43]. These sensors can also be used to detect atmospheric changes [51]. For example, barometric pressure sensors have the ability to detect changes in the atmosphere that are helpful for the prediction of weather patterns and changes. Another example is vacuum sensors, which are used when pressure in a vacuum is below atmospheric pressure levels, which can be difficult to detect using mechanical methods. Table 3 shows the different sub-types of pressure sensors along with their key features.

#### 3.1.3. Position Sensors

These sensors are used to sense the positions of valves, doors, throttles etc. These sensors are equipped with location tracking abilities that help to determine the precise positions of work-in-progress, tools, and other production-relevant items within the facility [57,58]. Motion sensors (which trigger actions such as illuminating a floodlight by detecting movement of an object) and proximity sensors (which detect that an object has come within the range of a sensor) are worth mentioning as they serve functions similar to position sensors [59,60]. Table 4 shows the different sub-types of position sensors along with their key features.

#### 3.1.4. Force Sensors

Force sensors are designated to translate applied forces (such as tensile, compressive force, etc.) into electric signals which reflect the degree of force [63,64]. These signals are then sent to indicators, controllers, or computers that inform operators about the processes, or serve as inputs that help to achieve control over machinery and processes. A variety of force sensors are being used in smart factories depending on the type of force being measured [65]. For instance, load cells measure compressive forces, strain gauges measure the internal resistance forces, and force sensing resistors measure the rate of change of an applied force. Table 5 shows the different sub-types of force sensors, along with their key features.

#### 3.1.5. Flow Sensors

These sensors have the ability to sense the movement of gases, liquids, or solids within a pipe or a conduit. These sensors have extensive uses in processing industries, and allow operation of the machinery at an optimum performance level [67,68]. A flow sensor can be electronic, using ultrasonic detection of the flow, or partially mechanical [69]. For instance, flow sensors in automobiles measure air intake in the engine and adjust fuel delivery to the fuel injectors in order to provide optimum fuel to the engine. Flow sensors are also used in medical ventilators, where the correct rate of delivery of air and oxygen to patients is needed for respiration [70]. Table 6 shows the different sub-types of flow sensors along with their key features.

### 3.2. Smart Sensors

**Figure 2:**
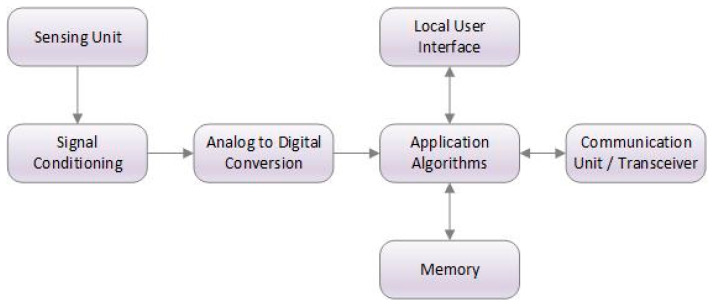
Smart sensor building blocks.

**Figure 3:**
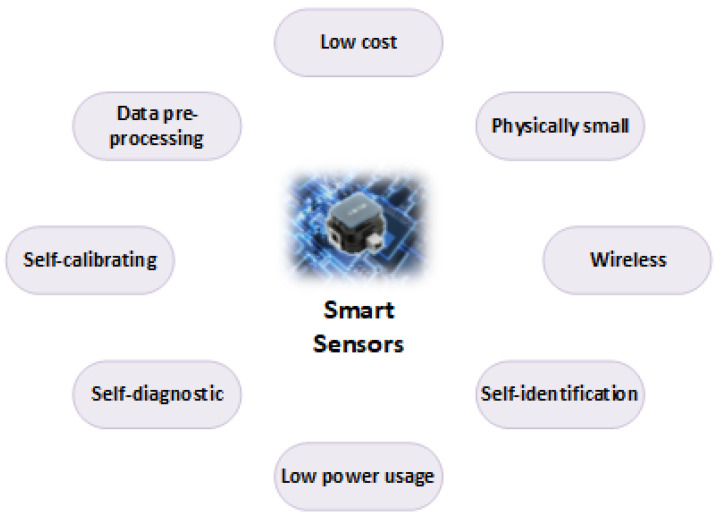
Features of a smart sensor.

Among other recent advances in technology, smart sensors have been in the spotlight in terms of their potential significance and wide range of application areas. With the integration of computing and IoT in industrial processes, ordinary sensors have been transformed into smart sensors providing them with abilities to carry out complex calculations with collected data [37,40]. Apart from increased capabilities, smart sensors have also become remarkably small and exceedingly flexible, turning bulky machines into high-tech intel. Equipped with signal conditioning, embedded algorithms, and digital interfaces, smart sensors have become devices with detection and self-awareness capabilities [25,72]. These sensors are built as IoT components that convert real-time information into digital data that can be transmitted to a gateway [36,73]. These abilities allow smart sensors to predict and monitor real time scenarios and take corrective actions in an instant. Complex multi-layered operations such as collecting raw data, adjusting sensitivity, and filtering, motion detection, analysis, and communication are the main functions of intelligent sensors [38]. For instance, wireless sensor networks (WSNs) are one of the applications of smart sensors, whose nodes are connected with one or more other sensors and sensor hubs, making a communication technology of some kind. In addition, information from multiple sensors can be combined to deduce conclusions about an existing problem; for instance, temperature and pressure sensor data can be used to infer the onset of a mechanical failure. Figure 2 shows the building blocks of a typical smart sensor [41], while Figure 3 summarizes the key features of smart sensors [51,72].

#### 3.2.1. Calibration Capability

The ability of a sensor to determine its normal function is termed calibration capability [39]. Self-calibration is simple in many cases, and different calibration techniques are available for different types of sensors:

- Sensors with an electric output carry out calibration by using a known reference of voltage level.
- Sensors such as load cells used for weighing systems can adjust their output to zero when no force is being applied [27].
- Other sensors can use look-up tables for calibration. However, to carry out calibration using look-up tables, a huge amount of memory capacity must be available to store correction points due to the large volume of data gathered during the process. On the contrary, an interpolation method is preferable in which a small matrix of correction points is required [35].

#### 3.2.2. Self-Diagnosis of Faults

Smart sensors carry out self-diagnosis by observing internal signals for evidence of faults. Differentiating between normal measurement deviations and sensor faults can be a challenge for some sensors [72]. This challenge is overcome by storing multiple measured values around a set-up point and calculating minimum and maximum values for the measured quantity [51]. In order to measure the impact of sensor fault on the measured quantity, uncertainty techniques are used. This enables the continuation of using a sensor after the fault has arisen.

### 3.3. Nuclear Sensors

Nuclear sensors are very uncommon [37,74] due to two reasons: they are costly and have strict safety regulations for their use. Recent developments have made the availability of low-level radiation sources for safe use of these sensors [37]. Table 7 summarizes the pros and cons of these sensors.

### 3.4. Micro-Sensors (MEMS Sensors)

Micro-sensors contain two and three dimensional micro-machined structures that are a part of microelectromechanical system (MEMS) devices [74,75]. These sensors can be regarded as small sized transducers, as they convert mechanical signals from an energy source into electrical form. Currently, sensors that measure temperature, pressure, force, speed, sound, magnetic field, optical, biomedical, and chemical features are being used successfully by industries [37,74]. Table 7 summarizes the main characteristics of these sensors.

### 3.5. Nano-Sensors (NEMS)

Nano-sensors, based on nanotechnology, are the most recent development in sensing technology [72,75]. These are a part of nano-elctromechanical system (NEMS) devices which include nano-actuators as well. Table 7 gives a summary of these sensors.

## 4. Conclusions

This paper has discussed the different types of sensor technology used in the manufacturing industry, specifically in smart factories. An extensive review of the extant literature is also presented on the technology lead smart factory. Key differences between a smart factory and traditional factory have been highlighted. Use of sensors, interoperability of different IoT devices, integration of robotics and AI, and use of VR techniques were found to be the key intelligent features of a smart factory. As sensors are an important component of a smart factory, the main sensor types and their sub-types, along with their characteristics, materials, uses, application areas, advantages and disadvantages have been identified in this paper. Sensors and Wi-fi are changing the way people communicate with the surrounding world, bringing a new era of connectivity, termed the internet of things. This technology has the potential to provide virtually boundless opportunities to businesses and communities, with enhanced connectivity and use of collected data. With the help of these technologies, data flow is integrated between partners, suppliers, and customers, as well as organizations to develop a finalized product according to customer demands. Manufacturers around the world are beginning to realize the importance of sensors, and the benefits of merging traditional operations and IoT. Therefore, a developing trend for the smart factory is human–machine collaboration. This paper attempted to provide a broad overview of the extant literature on Industry 4.0, summarizing the variations between traditional and smart factories, and the main sensors used within a smart factory setup.

While aspects of the smart factory have been successfully implemented in several key industries (e.g., the chemical industry), it has been found that there are technological, cost, and knowledge barriers to the broader implementation of sensors across manufacturing [20]. These barriers also include standardization, cyber security, and risk concerns, which may enhance organizational resistance to automation within the factory. In terms of standardization, two Industry 4.0 reference architectures have been standardized, namely, Reference Architecture Model Industry 4.0, from the International Electrotechnical Commission, and Industrial Internet Reference Architecture, from the Object Management Group. A discussion on these standardized architectures and the compatibility of each Industry 4.0 proposal to these standards is needed. Moreover, the concept of digital twin in the manufacturing system configuration stage, which can enable the validation of system performance in a semi-physical simulation manner, is gaining interest. The digital twin conducts a direct test and validation that can quickly locate the problem and malfunctioning part, rectify the design mistakes, and test the workability of equipment in the system execution [76,77]. In addition, a deficiency of labor skills and manufacturer technical willingness relative to available technologies can be limiting. It has been revealed that there is a scarcity of studies that address these issues. Furthermore, artificial intelligence, block chain, cloud computing, and big data analytics (also known as ABCD) are known for the generation of information technology [76]. The employment of blockchain in smart factories is known to enable increased security, enhanced traceability, and reduced costs [76,77]. These features along with standardization and cyber security issues need a detailed study and therefore, are identified as a future extension of this work.

## Figures and Tables

**Table 1:** Key differences between the traditional manufacturing factory and smart factory.

Traditional Factory	Smart Factory
Manual and isolated processes, operations; no integration with different systems and tools.	Digitized and integrated processes, operations; complete integration with existing systems, new systems and tools.
Legacy systems with frequent machine failures and increased maintenance costs.	Smart systems with improved machine utilization and reduced maintenance costs.
Tied to systems or machines for data, therefore zero or limited data for decision making; process-driven decision making.	Update or receive data on the go, therefore complete data for faster decision making; data-driven decision making.
Limited technology involvement.	Internet-of-things (IoT), sensor, mobile app, radio frequency identification (RFID) enabled.
Zero or limited visibility on operations, productivity data.	Increased transparency, visibility on operations and production data.
Limited innovation in production development.	Smart and intelligent products.
Inaccurate asset tracking process and poor resource utilization.	Accurate asset tracking using IoT, RFID; improved resource utilization.
Poor interoperability.	High interoperability.
The production line is fixed unless manually reconfigured by people with system power down.	When switching between different types of products, the needed resources and the route to link these resources should be reconfigured automatically and online.

**Table 2:** Key features of temperature sensors.

Sensor Types	Characteristics	Material	Uses	Advantages	Disadvantages	Source
Thermistor	Additionally called thermally sensitive resistors, change their physical appearance with changes in temperature. The effective operating range is −50 °C to 250 °C.	Ceramic such as oxides of nickel, manganese or cobalt coated in glass.	Widely used in automobile industry to detect the intake and coolant temperature.	Fast thermal response; lead wire resistance results in small error.	Limited temperature range; low resistance to shock.	[42,43,44,45,46,47,48,49,50]
Resistance Thermometer	Have a fixed relationship with temperature, resistance changes as temperature changes. Known for accuracy and stability, these sensors detect temperature changes ranging from −50 °C to 500 °C for thin film, and −200 °C to 850 °C for wide film.	High purity conducting metals such as platinum, copper or nickel wound into a coil.	Most widely used as HVAC, room, duct and refrigerant temperature, motors for overload protection and in automotives for air and oil temperature detection.	High precision and stability; strong output signal and high sensitivity; good stability (can maintain temperatures below 0.1 °C for a long time).	Expensive; easily influenced by lead wire resistance; slow thermal response; low resistance to shock and vibration.	
Thermocouple	Temperature changes cause a temperature dependent voltage which is in turn converted into a temperature reading. Detect temperatures as high as 3000 °C and as low as −250 °C.	Two junctions of different materials such as copper and constantan that are welded together.	Most widely used in industrial measurement due to inexpensive, rugged and reliable nature.	Wide temperature range; high temperature measurement; high resistance to shock and vibration; fast thermal response.	Compensating conductors needed when extending lead wires.	

**Table 3:** Key features of pressure sensors.

Sensor Type	Characteristics	Material	Uses	Advantages	Disadvantages	Source
Resonant	Follows the principle of vibrating wire, where a magnetic coil is attached to a diaphragm which vibrates when faced by a magnetic field conducting an electric current. The vibration frequency depends on pressure applied.	Metal resistive element such as silicon and quartz.	Used in industrial gauge and vacuum measurement.	High over-pressure and burst pressure capabilities.	Expensive; limited machining processes of quartz.	[48,52,53,54,55,56]
Capacitive	Most commonly used. Display change in capacitance when pressure is applied to the diaphragm creating an oscillator frequency.	Metals such as copper and indium tin oxide.	Ideal for flow applications.	Highly sensitive, can measure high and low changes; measure pressures below 10 mbar; withstand large overloads.	Material constraints and joining and sealing requirements restrict applications.	
Piezoelectric	Use the properties of piezoelectric materials such as quartz to create a charge which is proportional to the force applied on the surface when pressure is applied.	Piezoelectric materials such as quartz, rochelle salt, barium titanium, and tourmaline.	Widely used for dynamic pressure measurement in turbulence, blast and engine combustion, also used in some medical applications such as monitoring arterial pulse.	Measures fast changing dynamic pressures.	Need high impedance circuit; susceptible to noise; unable to measure solid state pressure due to dynamic nature.	
Optical	Use interferometry to detect changes in pressure in optical fiber. Can be created using tiny components or micro-electromechanical systems (MEMS) technology.	Use a Fabry-Perot interferometer, with two partially reflecting mirrors made of glass or quartz.	Most widely used in radiography equipment.	Not disturbed by electromagnetic interference, allowing use in noisy conditions; highly sensitive, small size, and long life span; medically safe for implantation.	Costly; susceptible to interference from environmental effects and physical damage.	

**Table 4:** Key features of position sensors.

Sensor Type	Characteristics	Material	Uses	Advantages	Disadvantages	Source
Potentiometric	Resistance-based sensors, use a resistive track with a wiper which moves with the movement of the object.	Carbon film.	Commonly used in computer game joysticks, steering wheels, industrial and robot applications.	Inexpensive and easy to use.	Wear easily due to moving parts; low accuracy, repeatability and limited frequency response; limited detection range due to small size of the wiper.	[57,59,60,61,62]
Capacitive	Consist of two plates separated by a dielectric material. Rely on detecting change in capacitance to measure the position of an object either by changing the dielectric constant or overlapping area.	Metallic electrodes used as plates, with a dielectric material between them.	Widely used in accelerometers, ice detection, spacing and thickness of materials.	Non-contact measurement; high resolution; can detect motion in both linear and angular directions; different material detection such as skin, plastic, metal, liquid, etc.	Sensitive to environmental changes such as humidity, temperature etc.	
Magnetostrictive Linear Position	Material changes its size or shape when in the presence of a magnetic field to detect the position of an object.	Ferromagnetic materials such as iron, nickel, and cobalt.	Used in the controlling of gaps between rollers, hydraulic or pneumatic cylinders, in automotive industry and electric actuators.	Non-contact; ability to detect position in the presence of a barrier between magnet and sensing rod; ability to measure multiple magnets with a single sensing rod.	Dead band on each side of the sensor cannot be reduced to zero.	
Eddy Current based	Work with induced currents that occur in a conductive material in the presence of a changing magnetic field using Faraday’s law of induction.	Conductive material such as copper, aluminum, titanium alloy etc.	Widely used in automation applications, machine tool mounting, final assembly of delicate machinery and monitoring drive shafts.	Functional in dirty environments; less expensive; unaffected by most contaminants.	Omnidirectional, can only determine the distance of the object not the direction of the object from the sensor.	
Optical	Work two ways: (1) light is transmitted from an emitter and sent to a receiver at the other end of the sensor; (2) emitted light signal is reflected from the monitored object towards the light source. Change in light characteristics are used to determine the position.	Glass or plastic disc used as an encoder, with LED used as a light source, and a photodetector as light receiver.	Widely used in deadbeat galvanometers, induction motors, induction furnaces, electric brakes, and speedometers.	Both linear and rotational movement can be detected.	Large amount of heat is produced in the soft core of transformers, induction coil, electric motors, etc., reducing the efficiency of these machines.	

**Table 5:** Key features of force sensors.

Sensor Type	Characteristics	Material	Uses	Advantages	Disadvantages	Source
Load cells	Convert applied force into an output signal measuring force such as compressive forces. Include pneumatic, hydraulic, piezoelectric crystal, inductive, capacitive, and magnetostrictive load cells.	Materials such as ferromagnetic, metal resistive elements, metallic electrodes etc.	Commonly used in truck scales.	Performance is affected by no-axial force; requires temperature network; excessive force may damage the load cells permanently.	Small and compact in size; good accuracy; less expensive; good sensitivity.	[58,63,64,65,66]
Strain gauges	Sensors whose electrical resistance changes with applied force.	An insulating substrate with a conductive metallic foil.	Widely used in load measuring applications, from truck scales to bolt tensioning devices.	High resolution; small size; measures both static and rapidly changing stress; low price.	Low accuracy; need to be calibrated after installation.	
Force Sensing Resistors (FSR)	Use a type of piezoresistive technology consisting of a semi-conductor material or ink sandwiched between substrates separated by a separator. A conductive film is formed with applied force and presses against a conductive ink printed on the substrate.	Electronic and electronic components; PCB, conductive foam.	Used in foot pronation systems, automobiles like car sensors, resistive touch pads, etc.	Thin and flexible; available in variety of sizes and shapes; low power consumption; low cost.	Low in precision and repeatability, repeated measurements vary by 10% or more.	

**Table 6:** Key features of flow sensors.

Sensor Types	Characteristics	Material	Uses	Advantages	Disadvantages	Source
Positive displacement	Perform direct measurement of volume of the fluid passing through the device. A known volume of fluid is trapped and moved through the sensor using rotating parts that effectively pass the fluid along sequentially before allowing more fluid to enter the device.	Stainless steel.	Used in measuring oils, gasoline, hydraulic fluids, and home installed metering of water and gas.	Function over a wide range of fluid viscosities; high accuracy; low maintenance requirements; provide mechanical or electronic interface.	Extremely expensive to install and maintain due to moving parts.	[46,67,68,69,70,71]
Mass flow	Detect energy transfer from a heated surface to a flowing fluid following different ways: (1) introducing thermal energy and measuring change in temperature; (2) maintaining constant temperature and measuring the amount of energy needed to do so; (3) introducing electric current to a resistive wire and measuring the current needed to maintain temperature.	Special alloys to cope with aggressive gases.	Widely used in automotive applications.	Directly measure liquid flow with high accuracy; wide range of measurable fluids, including highly viscous liquids; bidirectional flow measurement.	Poor zero stability; cannot measure liquids with low density; highly sensitive to vibration interference.	
Velocity flow	Sensors detect flow rate by measuring the velocity of fluid flowing through the sensor.					
	**Mechanical:** Fluid flow measured by the movement of a paddle wheel detected by a magnetic coil or infrared sensor.	Stainless steel.	Commonly used in water/waste treatment plants.	Cost effective; compact; need very little energy to operate; detect a wide variety of fluids.	Moving parts are subject to wear; build-up of contamination due to flow of dirty fluids; a minimum amount of fluid needed to move the paddle wheel.	
	**Electromagnetic:** Operate on Faraday’s law of induction. A coil induces a magnetic field in the fluid being measured and uses a set of electrodes to measure the induced voltage.	Hastelloy, tantalum 90% platinum 10%, iridium and titanium for electrodes.	Widely used in chemical manufacturing, petrochemical industries.	Can measure liquids with some degree of contamination; pressure drop is not induced in the pipe.	Do not function with non-conductive fluids; not suitable for vacuum conditions; require fluids to have some level of minimum conductivity.	
	**Ultrasonic:** A pair of ultrasonic transducers generate a signal directed into the fluid flow, each signal is directed back to the receiver using a set of mirrors.	Stainless steel pipe wall.	Used in facilities management, aquafarms, pulp and paper manufacturing.	Used for both conductive and non-conductive fluids; handle high temperatures and pressures; can be non-wetted.	Fluids with air bubbles cannot pass through ultrasonic energy; high vibrations cause difficulty in reading; high cost.	

**Table 7:** Key features of nuclear, micro, and nano sensors.

Sensor Type	Characteristics	Material	Uses	Advantages	Disadvantages	Source
Nuclear sensor	Operate following the principles of optical sensors, where a medium facilitates the transmission of radiation between a source and a detector; and the magnitude of transmission is attenuated according to the measured variable.	Cesium 137—as gamma ray source; sodium diode device as gamma ray detector.	Mass flow measurement and medical scanning applications.	Zero carbon emission; energy independence.	Very expensive; are prone to contamination by background radiation.	[74,75]
Micro-sensors	An element with some sort of mechanical functionality is integrated with microelectronics. The typical sizes of these sensors range between 0.01 mm or 10^–5^ m to 5 mm.	Silicon semiconductor material; sometimes fabricated with metals, plastics, polymers, gasses, and ceramics deposited on the silicon base.	Largely used in the automotive industry and medical equipment, such as blood pressure measurement.	Smaller size; improved performance; better reliability; lower production costs.	Have low capacitance; output signals prone to noise contamination; produce output signals of very low magnitude.	[74,75]
Nano-sensors	Vary in size from 1 to 1000 mm, using nanotechnology.	Thin layers of metal films or semiconductors; more advance than MEMS using special forms of etching, optical lithography or electron beam lithography.	Used as accelerometers, biological sensors and sensors for airborne chemicals.	Lower production costs; reduced power consumption; smaller size.	Complicate to handle; short-term noise issues.	[72,74]
